# Characterization of the complete chloroplast genome of *Hippophae salicifolia* D. Don (Elaeagnaceae)

**DOI:** 10.1080/23802359.2021.1934142

**Published:** 2021-05-31

**Authors:** Yiwei Tang, Ruoqiu Wang, Qiong La, Wenju Zhang

**Affiliations:** a School of Life Sciences, Ministry of Education Key Laboratory for Biodiversity Science and Ecological Engineering, Institute of Biodiversity Science, Fudan University, Shanghai, China; b Research Center for Ecology, College of Science, Tibet University, Lhasa, China

**Keywords:** *Hippophae salicifolia*, chloroplast genome, phylogenetic analysis

## Abstract

Seabuckthorn (*Hippophae* L.) is a pioneer species widely distributed in Eurasia. We assembled and annotated the chloroplast genome of *Hippophae salicifolia* from Illumina pair-end data, which was 155,420 bp in length with 36.74% GC content; this plastome featured a quadripartite structure with two copies of a large inverted repeat (IR) of 26,528 bp separated by large single copy (LSC) region of 83,504 bp and small single copy region (SSC) of 18,860 bp. In total, 131 complete genes were annotated, including 38 tRNA, eight rRNA, and 85 protein-coding genes. Phylogenetic analysis recovered *H. salicifolia* and *H. gyantsensis* as monophyletic and sister to all other Hippophae species for which complete plastome sequences have been published.

Genus *Hippophae* (Elaeagnaceae) consists of dioecious shrub or small tree species, distributed widely across Eurasia with a center of diversity in the Qinghai-Tibet Plateau (QTP) (Qin and Gilbert 2007). In northern China, seabuckthorn is widely used in sand fixation and preventing desertification. However, the phylogenetic relationships within *Hippophae* are controversial. Reconstructions based on five nuclear DNA regions supported a clade containing *H. neurocarpa* and *H. salicifolia*; based on five chloroplast genes; however, this clade has low support. Combined nuclear and plastid data resolved a clade composed of *H. tibetana* and *H. rhamnoides* with moderate to strong support (Jia and Bartish 2018). Nevertheless, the phylogenetic relationships among *H. neurocarpa*, *H. gyantsensis*, and *H. tibetana* based on 32 complete chloroplast genomes revealed *H. rhamnoides* was more closely related to *H. neurocarpa* than *H. tibetana* (Wang et al. 2018; Zhou et al. 2019; Zhou et al. 2020). Here, we sequenced and assembled the complete chloroplast genome of *H. salicifolia*, and compared with other *Hippophae* species.

The specimen of *H. salicifolia* was collected on the QTP in China (N 27.868339, E 91.800321). The voucher specimen was deposited in the herbarium of School of Life Sciences Fudan University (herbarium code: FUS, voucher number: HT0334, contact person: Ruoqiu Wang, email: 19110700126@fudan.edu.cn). Fresh leaves were used to extract total genomic DNA (gDNAs) with Plant Genomic DNA Kit (Tiangen Biotech Co., Beijing, China). The 350 bp paired end library was constructed according to the manufacturer’s introductions (Illumina, San Diego, CA) and sequenced on Illumina Hiseq 2500 platform (Illumina, San Diego, CA) for 150 bp paired-end sequencing. We obtained a data set containing a total of 32.6 G bp. To avoid reads with artificial bias (i.e. low quality paired reads, which primarily result from base calling duplicates and adaptor contamination), we removed the following types of reads: (i) reads with 3 nt unidentified nucleotides (N); (ii) reads aligned to the adaptor; (iii) reads with ≥20% bases having Phred quality ≥5. The complete chloroplast genome was assembled with GetOrganelle v1.7.2a (Jian et al. 2019; Luo et al., 2012), and the average read coverage was 4661× (range of read coverage: 29–7869×). GapCloser v2.04 (Luo et al. 2012) was used to fill gaps and MUMmer v3.23 (Kurtz et al. 2004) was used for alignment. The DOGMA tool (Dual Organellar GenoMe Annotator; http://dogma.ccbb.utexas.edu/) was used for annotation (Wyman et al. 2004). The complete *H. salicifolia* chloroplast genome was submitted to NCBI (https://www.ncbi.nlm.nih.gov, GenBank accession number: MW392804). Complete chloroplast genome sequences were aligned using MAFFT (version 7.452) (Katoh et al. 2019) and adjusted manually. Phylogenetic relationships among *H. salicifolia* and other Elaeagnaceae species were reconstructed with maximum likelihood using RAxML (Stamatakis 2014).

With this alignment of complete chloroplast genomes for several species of *Hippophae* and *Elaeagnus*, we found that, the gene content, average CG content and intron content of *Hippophae* are comparable to that of *Elaeagnus* (51.09% vs. 50.82%, 36.68% vs. 37.07%, 6.83% vs. 6.57%, respectively). However, we cannot find any genes or intergenic spacers that are particularly hyper-variable within *Hippophae* or *Elaeagnus* that could be used for species discrimination. The effective number of codons (ENC) of *Hippophae* and *Elaeagnus* chloroplast genomes coding sequence was 49.42–50.58 and 49.19–49.77, respectively, indicating a slightly weaker of codons bias in *Elaeagnus*.

Phylogenetic analysis recovered all seven *Hippophae* species as monophyletic with a clade containing *H. salicifolia* and *H. gyantsensis* as sister to the rest. All *H. rhamnoides* subspecies clustered in a well-supported clade (BP = 100%). Notably, *H. neurocarpa* nested into subclade *H. rhamnoides* (BP = 100%) (Figure 1), which was inconsistent with the previous study (Jia and Bartish 2018). We speculate that the sample of the sequence NC_047483 does not belong to *H. neurocarpa* but to the hybrid of *H. rhamnoides* ssp. *sinensis* and *H. neurocarpa*, because the sample (NC_047483) was collected in the hybrid zone between *H. rhamnoides* ssp. *sinensis* and *H. neurocarpa*, and the hybrid individuals possess transitional morphology between their parents (Du et al. 2008). Incorrect identification or sampling error of *H. neurocarpa* (NC_047483) may led to this confusing result.

**Figure 1.**
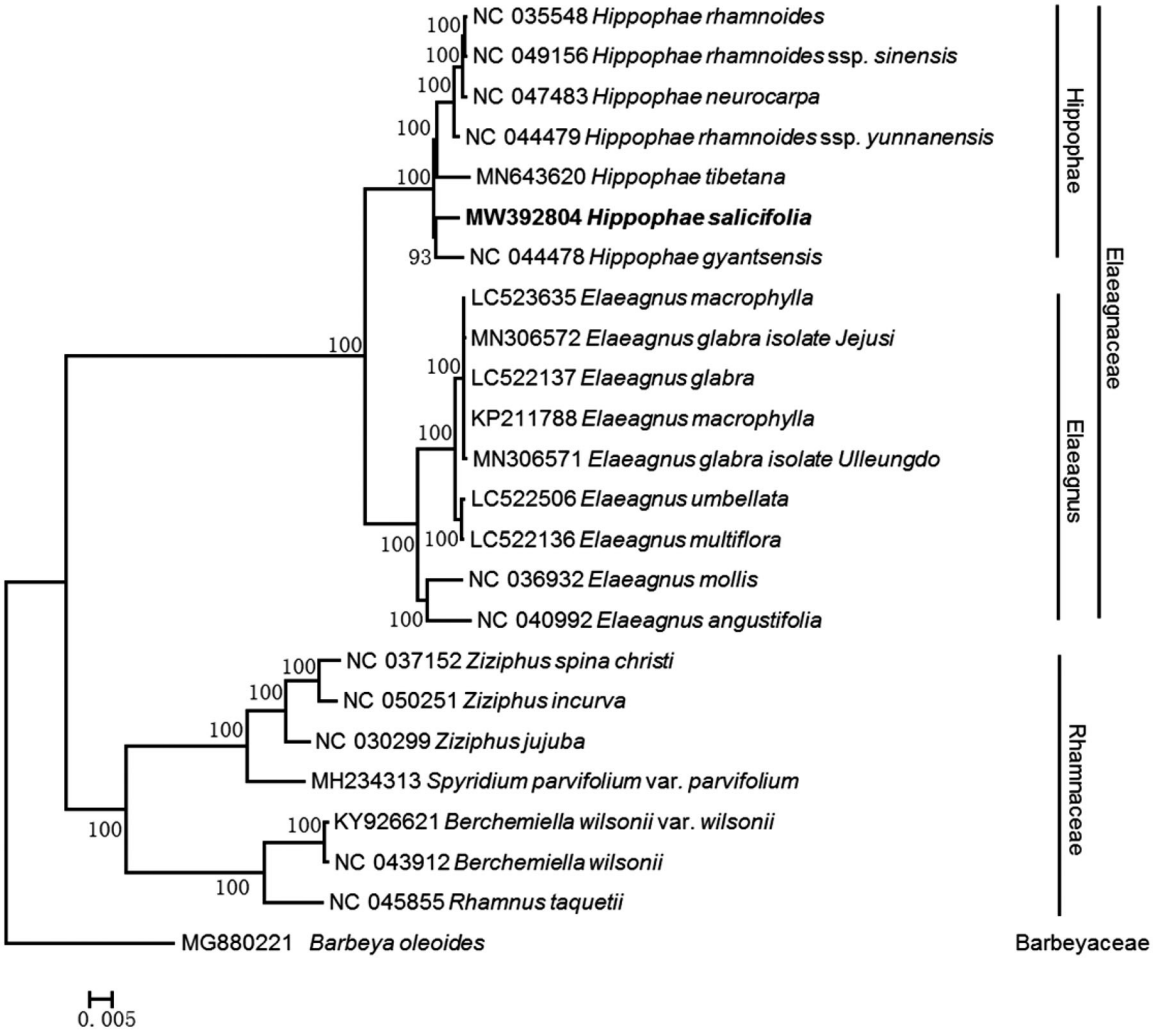
Maximum-likelihood phylogenetic tree based on the complete chloroplast genome of 24 species. Numbers at nodes indicate bootstrap support values (1000 replicates). The numbers on branches are bootstrap support values.

## Data Availability

The genome sequence data that support the findings of this study are openly available in GenBank of NCBI at https://www.ncbi.nlm.nih.gov under the accession no. MW392804. The raw sequence data used in this research were deposited successfully with registered numbers of associated BioProject, SRA, and Bio-Sample: PRJNA689712, SRR13357448, and SAMN17215125, respectively.
